# Genetic characterization of Shiga toxin-producing *Escherichia coli* O26:H11 strains isolated from animal, food, and clinical samples

**DOI:** 10.3389/fcimb.2015.00074

**Published:** 2015-10-20

**Authors:** Alejandra Krüger, Paula M. A. Lucchesi, A. Mariel Sanso, Analía I. Etcheverría, Ana V. Bustamante, Julia Burgán, Luciana Fernández, Daniel Fernández, Gerardo Leotta, Alexander W. Friedrich, Nora L. Padola, John W. A. Rossen

**Affiliations:** ^1^Laboratorio de Inmunoquímica y Biotecnología, Facultad de Ciencias Veterinarias, Centro de Investigación Veterinaria de Tandil, Consejo Nacional de Investigaciones Científicas y Técnicas, Comisión de Investigaciones Científicas, Universidad Nacional del Centro de la Provincia de Buenos AiresTandil, Argentina; ^2^Línea Seguridad Alimentaria, Instituto de Genética Veterinaria Ing. F.N. Dulout, Consejo Nacional de Investigaciones Científicas y TécnicasLa Plata, Argentina; ^3^Department of Medical Microbiology, University Medical Center Groningen, University of GroningenGroningen, Netherlands

**Keywords:** Shiga toxin-producing *Escherichia coli*, O26:H11, genetic characterization, microarray, MLVA, hemolytic uremic syndrome, cattle

## Abstract

The Shiga-toxin producing *Escherichia coli* (STEC) may cause serious illness in human. Here we analyze O26:H11 strains known to be among the most reported STEC strains causing human infections. Genetic characterization of strains isolated from animal, food, and clinical specimens in Argentina showed that most carried either *stx*_1a_ or *stx*_2a_ subtypes. Interestingly, *stx*_2a_-positive O26:H11 rarely isolated from cattle in other countries showed to be an important proportion of O26:H11 strains circulating in cattle and food in our region. Seventeen percent of the isolates harbored more than one gene associated with antimicrobial resistance. In addition to *stx*, all strains contained the virulence genes *eae*-β, *tir, efa, iha, esp*B, *cif*, *esp*A, *esp*F, *esp*J, *nle*A, *nle*B, *nle*C, and *iss*; and all except one contained *ehxA, esp*P, and *cba* genes. On the other hand, *tox*B and *esp*I genes were exclusively observed in *stx*_2_-positive isolates, whereas *kat*P was only found in *stx*_1a_-positive isolates. Our results show that O26:H11 STEC strains circulating in Argentina, including those isolated from humans, cattle, and meat products, present a high pathogenic potential, and evidence that cattle can be a reservoir of O26:H11 strains harboring *stx*_2a_.

## Introduction

Shiga toxin-producing *Escherichia coli* (STEC) are *E. coli* strains that can cause human diseases, like gastrointestinal illnesses and hemolytic uremic syndrome (HUS). *E. coli* O157 has been the most commonly reported STEC serogroup since it was identified in the 1980s. However, the number of non-O157 STEC infections has increased substantially in the last years. The O26 is one of the few serogroups frequently reported in non-O157 cases (Rivas et al., 2010; EFSA, 2013; Gould et al., 2013; ISPCH, 2014). In particular, strains of serotype O26:H11/- have been associated with severe human diseases (Gerber et al., 2002; Zimmerhackl et al., 2010; Käppeli et al., 2011).

All STEC strains are characterized by the ability to produce Shiga toxins (Stx). The Stx family consists of two major types: Stx1 and Stx2, which can be further divided into several subtypes. A single STEC strain may carry one or more Shiga toxin-encoding genes (*stx*) which are generally carried by prophages. Epidemiological studies indicate that different subtypes of *stx* are related to different clinical manifestations after STEC infection (Krüger and Lucchesi, 2015). Particularly, the *stx*_2a_ subtype is associated with highly virulent strains and HUS. Characterization of *stx* genotypes showed that STEC O26 strains isolated from patients can harbor *stx*_1a_, *stx*_2a_ or both, however, strains harboring only *stx*_2a_ were significantly associated with HUS (Bielaszewska et al., 2013). Furthermore, Bielaszewska et al. (2013) identified a new highly virulent *stx*_2a_-positive O26 clone as an emerging cause of HUS in Europe.

The production of Stx seems to be essential but not solely responsible for STEC pathogenicity. Other known and putative virulence factors are usually present in pathogenic STEC strains. Some of them, like the adhesin intimin encoded on a bacterial chromosomal pathogenicity island and the enterohemolysin EhxA encoded on a plasmid have been found in association with severe clinical disease in humans (Boerlin et al., 1999; Aldick et al., 2007).

It is a well-known fact that ruminants are the main reservoir of STEC strains (Naylor et al., 2005). Some studies suggest that domestic animals serve as reservoir for human pathogenic O26 STEC strains (Leomil et al., 2005). The O26 STEC strains also seem to be widely distributed in cattle, since those strains have been isolated from bovines belonging to different animal categories and production systems (Monaghan et al., 2011; Fernández et al., 2012; Paddock et al., 2014; Bonardi et al., 2015; Ison et al., 2015). Moreover, O26:H11 strains have been isolated from beef and dairy products (Bosilevac and Koohmaraie, 2011; Madic et al., 2011; Mohammed et al., 2014). Several studies from Europe and the United States report that O26 strains isolated from food and cattle generally carry *stx*_1_ or both *stx*_1_ and *stx*_2_ genes; moreover, O26 strains harboring only *stx*_2a_ have been rarely isolated from cattle and food (Pearce et al., 2006; Geue et al., 2009; Bonanno et al., 2015; Ison et al., 2015).

Our aim was to characterize O26:H11 STEC strains isolated from cattle, food and humans to contribute to the global knowledge of virulence profiles and epidemiology of O26 strains circulating in Argentina.

## Material and methods

### Bacterial strains and growth conditions

*E. coli* O26:H11 strains were selected from STEC collections in Argentina. The strains had been isolated between 1995 and 2013, from cattle, meat, human, and farm environment (Table 1). Most of the strains had been previously characterized by PCR regarding the presence of *stx*_1_, *stx*_2_, *eae, ehxA*, and *saa* genes. Strains were stored at −70°C with 20% (v/v) glycerol and when necessary grown in Luria Bertani broth at 37°C overnight.

**Table 1:** **Characteristics of O26:H11 STEC isolates tested in this study**.

**Strain**	**Period of isolation**	**Source**	**References**
1	2009	Milk-fed calf, Farm E	Fernández et al., 2012
2	2009–2010	Ground, Farm E	Polifroni et al., 2012
3	2009	Milk-fed calf, Farm E	Fernández et al., 2012
4	1995–1996	Calf	Blanco et al., 2004
5	1995–1996	Calf	Blanco et al., 2004
6	1995–1996	Calf	Blanco et al., 2004
7	1995–1996	Calf	Blanco et al., 2004
8	1995–1996	Calf	Blanco et al., 2004
9	1995–1996	Calf	Blanco et al., 2004
10	2002–2009	Human with diarrhea	Rivero et al., 2010
11	2002–2009	Human with diarrhea	Rivero et al., 2010
12	2009	Milk-fed calf, Farm D	Fernández et al., 2012
13	2009	Milk-fed calf, Farm E	Fernández et al., 2012
14	2010	Newborn calf, Farm A	Fernández et al., 2012
15	2010	Newborn calf, Farm A	Fernández et al., 2012
16	2010	Newborn calf, Farm D	Fernández et al., 2012
17	2009–2010	Cow, Farm A	Fernández et al., 2012
18	2009	Milk-fed calf, Farm E	Fernández et al., 2012
19	2010	Newborn calf, Farm A	Fernández et al., 2012
20	2010	Newborn calf, Farm B	Fernández et al., 2012
21	2010	Newborn calf, Farm B	Fernández et al., 2012
22	2009	Growing calf, Farm D	Fernández et al., 2012
23	2010	Human with diarrhea	This study
24	2009	Human with diarrhea	This study
25	2009	Cow	This study
26	2013	Beef	This study
27	2013	Beef	This study
28	2013	Ground meat	This study
29	2009	Calf feed, Farm E	This study

### Microarray-based characterization of virulence factors

Bacterial genomic DNA was extracted with the UltraClean Microbial DNA Isolation Kit (Mo Bio) or Wizard Genomic DNA Purification Kit (Promega) according to the manufacturer's instructions. Virulence and antimicrobial resistance genes were evaluated with a commercial oligonucleotide microarray for *E. coli* according to the manufacturer's protocol (CLONDIAG *Escherichia coli* combined Assay, Alere Technologies GmbH; Geue et al., 2010). The array contained 87 probes targeting virulence genes and 102 probes targeting antimicrobial resistance associated genes. Visualization of hybridization was achieved using the ArrayMate instrument (CLONDIAG GmbH) and signals were analyzed automatically. The results were converted into a binary numerical format (1– present, 0– absent) and further analyzed using BioNumerics (Version 6.6; Applied Maths).

### *stx* subtyping

Specific PCR reactions were performed to identify *stx*_1a_, *stx*_1b_, and *stx*_1c_ subtypes (Scheutz et al., 2012).

The presence of *stx*_2b_, *stx*_2e_, *stx*_2f_, and *stx*_2g_ subtypes was evaluated with the oligonucleotide microarray. This assay also detects *stx*_2a_, *stx*_2c_, and *stx*_2d_ subtypes but does not discriminate among them. Therefore, strains positive with the probe that detects *stx*_2a, c, d_ subtypes where further subtyped with specific PCR reactions (Scheutz et al., 2012).

### Detection of *eae*-β gene

Strains were tested for the presence of the *eae*-β subtype by specific PCR using the primer set SK1/LP4 (Oswald et al., 2000).

### Antibiotic susceptibility testing

Isolates carrying resistance genes were tested for resistance to several antimicrobials by the disk diffusion method according to the guidelines of the Clinical and Laboratory Standards Institute (CLSI, 2014). The following antimicrobial disks were used: ampicillin (10 μg), cephalothin (30 μg), cefotaxime (30 μg), cefoxitin (30 μg), amoxicillin/clavulanate (20 μg-10 μg), gentamicin (10 μg), amikacin (30 μg), streptomycin (300 μg), nalidixic acid (30 μg), ciprofloxacin (5 μg), chloramphenicol (30 μg), trimethoprim-sulfamethoxazole (1.25–23.75 μg), nitrofurantoin (300 μg), tetracycline (30 μg), colistin (10 μg), florfenicol (30 μg).

### Multiple-locus variable number tandem repeat analysis (MLVA)

For typing, an MLVA protocol that comprises nine variable number of tandem repeats (VNTR) loci, seven loci proposed by Lindstedt et al. (2007), i.e., CVN001, 002, 003, 004, 007, 014, 015, and two additional loci proposed by Løbersli et al. (2012), i.e., CVN016 and 017 was used. The seven loci proposed by Lindstedt et al. (2007) were amplified as described by Bustamante et al. (2010) and the two additional ones according to González et al. (2014). PCR products were analyzed in 6% denaturing polyacrylamide gels, and detected by silver staining (GenePrint STR Systems—Silver Stain Detection, Promega).

Allelic variants identified for each VNTR were sequenced with the same primers used to amplify those regions (Macrogen, Inc.). The sequences obtained were analyzed using Chromas 2.32 software (Technelysium Pty. Ltd.) and allele sequences of each VNTR were aligned with the software Clustal W (Larkin et al., 2007) in order to identify the number of tandem repeat units (TR). Alleles were named according to the number of TR. The absence of an amplification product was considered a null allele (−2).

The diversity index (D_*N*_), based on Nei's marker diversity, was calculated for each locus using the formula D_*N*_ = 1–Σ(fra)^2^, where fra is the allelic frequency (Noller et al., 2003). The discriminatory power of the method was assessed using the Simpson diversity index (D_*S*_) (Hunter and Gaston, 1988).

## Results

### Genetic characterization of virulence factors

**Figure 1:**
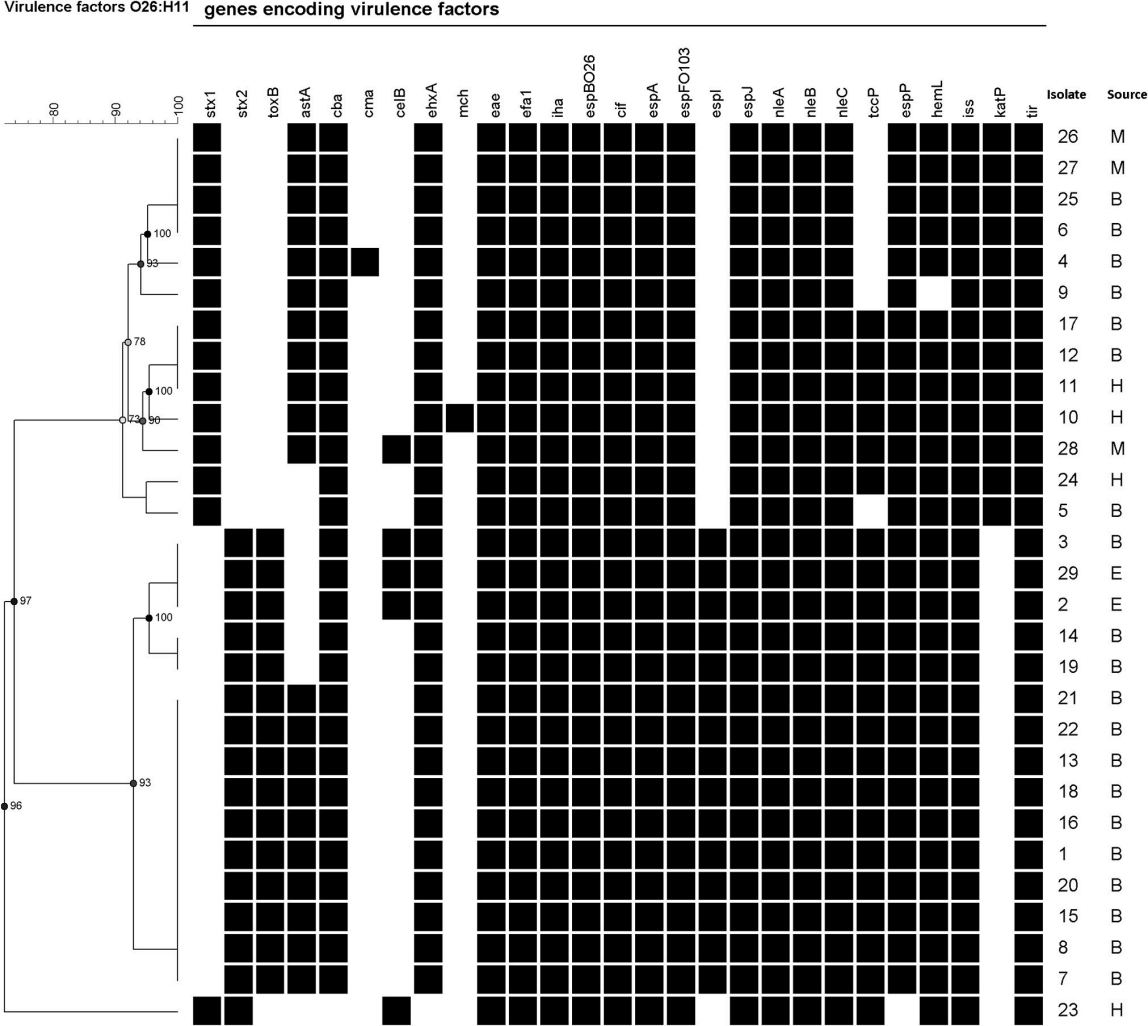
**Cluster analysis of genes associated with virulence**. B, E, H, and M indicate isolates obtained from bovine, environmental, human, and meat sources, respectively. Results for the *lpf* gene are not included because of the ambiguous signal observed for all strains. Genes not found in the any of the studied strains: *bfp*A, *ccl, cdt*B, *cfa*C, *cnf* 1, *cof* A, *eaa*A, *eat*A, *epe*A, *esp*A_C_rodentium, *esp*B_O157, *esp*C, *esp*F, *esp*F_C_rodentium, *etp*D, *fan*A, *fas*A, *fed*A, *fed*F, f17-A, f17-G, *fim*41a, *hly*E, *ipa*D, *ipa*H, *ire*A, *iro*N, K88ab, *lng*A *ltc*A, *nfa*E, *nle*B O157:H7, *nle*B Salmonella, *per*A, *pet, pic, prf* B, *rpe*A, *saa, sat, sen*B, *sep*A, *sfa*S, *sig*A, *sta*1, *sta*2, *stb, stx*_2b_, *stx*_2c_, *stx*_2d_, *stx*_2e_, *stx*_2f_, *stx*_2g_, *sub*A, *tsh, vat, vir*F.

Figure 1 shows the genes encoding virulence factors detected in the O26:H11 strains, clustered according to the Bionumerics analysis. Genes that were not found in any isolate are summarized in a footnote in the figure.

The *stx* types identified with the array were in agreement with previous PCR results. Forty five percent of the isolates were *stx*_1_-positve, 52% *stx*_2_-positive, and one isolate (3%) was *stx*_1_ and *stx*_2_-positive. By using the PCR subtyping protocol, all *stx*_1_ genes corresponded to the *stx*_1a_ subtype and all *stx*_2_ corresponded to the *stx*_2a_ subtype.

All tested isolates harbored the genes encoding for the adhesin Intimin (*eae* subtype β), and its receptor Tir (*tir*). Other adhesion related genes were also identified. The presence of *efa*1, *esp*B, and *iha* genes was demonstrated in all isolates, whereas *esp*P was found in all except the *stx*_1_ and *stx*_2_-positive isolate. The *tox*B gene was only observed in *stx*_2_-positive isolates. The *fas*A, *fed*A, *fed*F, *fim*41a, *nfa*E, and *saa* genes were not found.

Among toxin-encoding genes, *ehxA* (encoding for a hemolysin) was present in 97% of the isolates and *ast*A (encoding for EAST1, the enteroaggregative *Escherichia coli* heat-stable enterotoxin 1) was detected in 72% of the isolates. The *cba* and *cel*B genes associated with colicin activity were identified in 97 and 17% of the isolates, respectively. Also, one bovine isolate was positive for the gene encoding colicin M (*cma*) and one isolate obtained from human with diarrhea was positive for *mch*B, *mch*C, *mch*F, and *mcm*A microcin genes.

Several type III secretion system components (translocators and effectors) were identified. All the strains were positive for *cif*, *esp*A, *esp*fO103, *esp*J, *nle*A, *nle*B, and *nle*C genes, and 76% of the isolates harbored *tcc*P. The *esp*I gene was only identified in *stx*_2_-positive strains. The *etp*D, a gene encoding for a type II secretion pathway-related protein, was not found.

The *iss* and *hem*L genes were identified in 100 and 97% of the isolates, respectively. The *kat*P gene, encoding a catalase peroxidase, was detected in *stx*_1_-positive isolates only.

### Antimicrobial resistance

Five O26:H11 isolates (17%) carried more than two antimicrobial resistance genes, being *bla*_TEM_, *str*A-*str*B, and *sul*2 the most frequently detected (Table 2). Genes that were not found in any of the isolates are summarized in a footnote in the table. The integron integrase encoding gene (*int*I1) was detected in isolate 21. The antimicrobial susceptibilities of the five isolates carrying resistance genes were determined using disk diffusion method. Four isolates were resistant to multiple antimicrobial agents (Table 2), and most genetic resistances were phenotypically confirmed. The exception was isolate 23 (human isolate) that carried *str*A, *str*B, and *sul*2 genes but was susceptible for the tested antibiotics.

**Table 2:** **Antibiotic resistance profiles present in O26:H11 STEC isolates**.

**Isolate**	**Source^a^**	**Genotypic resistance profile^b^**	**Phenotypic resistance profile^c^**
4	B	*bla*_TEM_ *tet*B	AMP_(R)_ AMC_(R)_ CEF_(I)_ TET_(R)_
21	B	*bla*_TEM_ *str*A *str*B *aad*A1 *tet*A *drf*A1 *sul*1 *sul*2 *floR*	AMP_(R)_ AMC_(I)_ CEF_(I)_ TET_(R)_ SXT_(R)_ CMP_(R)_ FFC_(R)_
23	H	*str*A *str*B *sul*2	
26	M	*bla*_TEM_ *str*A *str*B *tet*B *sul*2	AMP_(R)_ AMC_(I)_ CEF_(I)_ S_(R)_ TET_(R)_ NAL_(R)_ SXT_(R)_
27	M	*bla*_TEM_ *str*A *str*B *tet*B *sul*2	AMP_(R)_ AMC_(I)_ CEF_(I)_ S_(R)_ TET_(R)_ NAL_(R)_ SXT_(R)_

a *B, H, and M indicate isolates obtained from bovine, human, and meat sources, respectively*.

b *The bla_TEM_ gene is associated with beta lactam resistance; strA, strB, and aadA1 with aminoglycoside resistance; tetA and tetB with tetracycline resistance; sul1 and sul2 with sulfonamide resistance; and drfA1 with trimethoprim resistance. Resistance genes studied but not detected in any of the studied isolates: vatE for streptogramin A resistance; aac_aph, aac3, aac3Ia, aac3Iva, aac6, aac6Ib, aadA2, aadA4, aadB, ant2, aphA, grm, armA, rmtA, rmtB, rmtC, rmtD, and npmA for aminoglycoside resistance; bla_ACC_, bla_ACT_, bla_CMY_, bla_KHM_, bla_MOX-CMY9_, bla_CTX-M1_/bla_CTX-M15_, bla_CTX-M2_/bla_CTX-M8_/bla_CTX-M26_, ctx_M9_, bla_DHA-1_, bla_FOX_, bla_GES-1_, bla_GIM-1_, bla_IMI-3_, bla_IMP_, bla_KPC-4_, bla_LAP-1_, bla_LEN-1_, bla_MOX_, bla_OXA-1_, bla_OXA-2_, bla_OXA-7_, bla_OXA-9_, bla_OXA-23_, bla_OXA-40_, bla_OXA-48_, bla_OXA-51_, bla_OXA-54_, bla_OXA-55_, bla_OXA-58_, bla_OXA-60_, bla_PER-1_, bla_PER-2_, bla_PSE-1_, bla_SHF-1_, bla_SHV_, bla_SME-1_, bla_SPM-1_, bla_VEB-1_,and bla_VIM_ for beta lactam resistance; ble for bleomycin resistance; catA1, catB3, catB8, catIII, and cmlA1 for chloramphenicol resistance; ereA and ereB for erythromycin resistance; ermB, mphA, and mrx for macrolide resistance; qepA, qnrA1, qnrB, qnrD, and qnrS for quinolone resistance; arr for rifampin resistance; sph for streptomycin resistance; sul3 for sulphonamide resistance; tet37, tetC, tetD, tetE, tetG, and tetX for tetracycline resistance; dfrA5, dfrA7, dfrA12, dfrA13, dfrA14, dfrA15, dfrA17, dfrA19 for trimethoprim resistance*.

c *Resistent (R) and intermediate (I) results for ampicillin (AMP), amoxicillin/clavulanate (AMC), cephalothin (CEF), cefotaxime (CTX), cefoxitin (FOX), gentamicin (GEN), amikacin (AKN), streptomycin (S), nalidixic acid (NAL), ciprofloxacin (CIP), chloramphenicol (CMP), trimethoprim-sulfamethoxazole (STX), nitrofurantoin (NIT), tetracycline (TET), colistin (COL), florfenicol (FFC)*.

### MLVA typing

**Figure 2:**
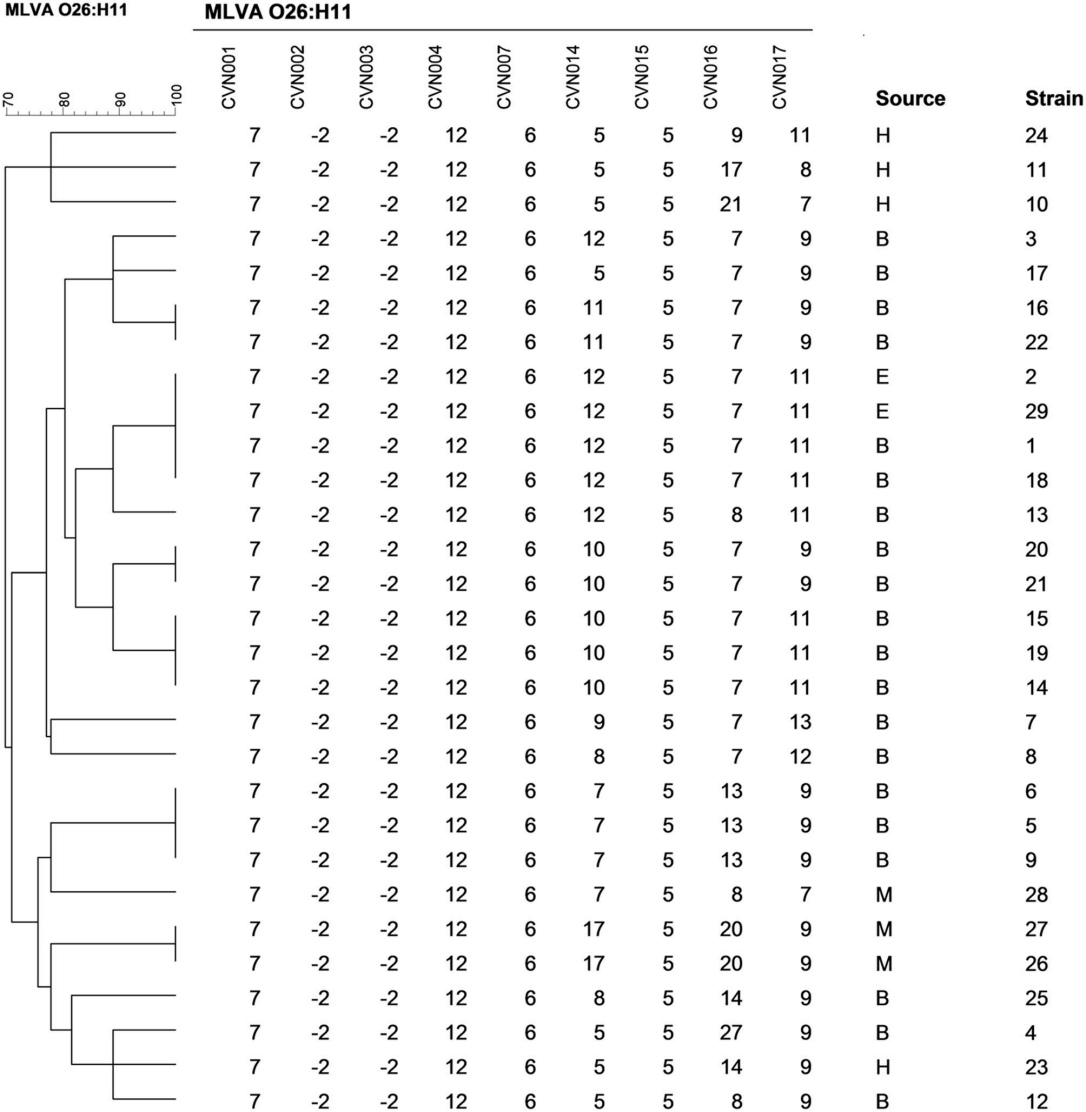
**Multiple-locus variable-number tandem repeat analysis–based clustering of STEC O26:H11 isolates investigated in this study**. Similarities among MLVA profiles were calculated using categorical coefficients and UPGMA clustering method. B, E, H, and M indicate isolates obtained from bovine, environmental, human, and meat sources, respectively.

The isolates could be principally discriminated by alleles of loci CVN014 (D_N_ = 0.83) and CVN016 (D_N_ = 0.70), followed by those of CVN017 (D_N_ = 0.63). On the other hand, loci CVN002 and CVN003 showed a null allele in all the isolates. MLVA typing divided the total number of strains into 19 distinct genotypes, 13 of them being unique (Figure 2). The Simpson's index of diversity, calculated for the combined typing set, showed a value of D_S_ = 0.96. Three main clusters were obtained: one group included the *stx*_2_- positive isolates and one *stx*_1_-positive isolate (isolate 17); a second group, most of the *stx*_1_-positive strains and the *stx*_1_ and *stx*_2_-positive isolate (isolate 23) and a third group included three s*tx*_1_-positive strains.

Isolates with the same MLVA type may indicate they are clonal. For some isolates this was supported by revealing a time-spatial relationship and by their identical virulence profile (isolates 14 and 19 from dairy farm A; 26 and 27 from a same meat processing plant; 20 and 21 from dairy farm B; 16 and 22 from dairy farm D; 1 and 18 from dairy farm E; and 2 and 29 also from dairy farm E).

## Discussion

STEC O26:H11 strains are considered among the most reported STEC strains involved in human infections worldwide. To our knowledge, there is no specific study of O26:H11 STEC strains in Latin America. Our aim was to determine the characteristics of *E. coli* O26:H11 strains circulating in Argentina in the period 1995–2013 to contribute to the global characterization of these strains.

Subtyping of *stx* genes indicated that O26:H11 strains in our region mainly present either *stx*_1a_ or *stx*_2a_ genotypes, similar to O26:H11 strains reported in other countries (Bielaszewska et al., 2013; Bletz et al., 2013; Bonanno et al., 2015). The *stx*_2d_ subtype, present in the new pathogenic O26:H11 clone circulating in France (Delannoy et al., 2015), was not detected in our collection.

Genetic characterization showed that in addition to *stx* genes, O26:H11 strains harbored genes encoding other toxins, adhesins, and components related to the type III secretion system that contribute to their virulence. In particular, *eae*-β, *tir, efa, iha, esp*B, *cif*, *esp*A, *esp*F, *esp*J, *nle*A, *nle*B, *nle*C, and *iss* genes were detected in all the isolates; and all except one contained *ehxA, esp*P, and *cba* genes.

The analysis based on the presence/absence of genes associated with virulence identified three main clusters, one containing the *stx*_1a_-positive isolates, another the *stx*_2a_-positive isolates and a third one the *stx*_1a_ and *stx*_2a_-positive isolate (Figure 1). The *tox*B, *esp*I, and *kat*P genes were differentially distributed between *stx*_1a_-positive and *stx*_2a_-positive groups. The *tox*B and *esp*I genes, previously associated with severe disease (Mundy et al., 2004; Michelacci et al., 2014), were exclusively present in *stx*_2a_-positive isolates. Conversely, the *kat*P gene encoding for catalase-peroxidase was only detected in the *stx*_1a_-positive isolates.

Heterogeneity in gene composition of STEC O26:H11 plasmids has been detected by Zhang et al. (2000). Considering the plasmidic markers *ehxA, kat*P, *esp*P, and *etp*D, two major subgroups of *stx*_2a_-positive strains were identified in Europe by Bielaszewska et al. (2013), one of them typical for the new virulent German clone. Noticeably, all the *stx*_2a_-positive strains of our study present a distinct profile, positive for *ehxA* and *esp*P, but negative for *kat*P and *etp*D genes. Thus, *stx*_2a_-positive strains of our Argentinian collection differ from the German clone, and also from the human-pathogenic strain recently identified in France (Delannoy et al., 2015), which is negative for all these plasmidic markers. The plasmid profile of *stx*_1a_-positive strains of our study (positive for *ehxA, kat*P, *esp*P, and negative for *etp*D) is the same that Zweifel et al. (2013) identified in the human *stx*_1_-positive strains from patients with HUS or bloody diarrhea in Switzerland. On the other hand, the unique *stx*_1_ and *stx*_2_-positive isolate was negative for all plasmidic genes (*tox*B, *esp*P, *kat*P, *ehxA, etp*D, *saa, sub*AB, *epe*A) tested, suggesting the absence of a virulence plasmid like pO26-Vir, pO157, or pO113.

The MLVA profiles found in the present study do not coincide with any of the profiles identified by Brandal et al. (2012) among ovine and human O26:H11 isolates from Norway. Although loci CVN001, 004, and 007 were monomorphic in both studies, the alleles were different. The loci CVN002 and CVN003 presented a null allele for all tested samples in our study, in agreement with results obtained by Løbersli et al. (2012) for O26 isolates. Available epidemiological information suggests that isolates forming clusters with identical MLVA profiles were derived from the same source and constitute a single clone. However, one cluster contained human *stx*_1_-positive isolates not epidemiologically related. In addition, two isolates (isolates 20 and 21) from the same farm had the same virulence and MLVA profiles but only isolate 21 had antimicrobial resistance genes. Although there is a possibility that these isolates are from the same clone and that isolate 21 acquired antimicrobial genes, it is also possible that MLVA typing is not discriminatory enough to distinguish both isolates from each other. Interestingly, major groups obtained by MLVA analysis were similar to those obtained by the analysis of virulence factors.

Antimicrobial resistance genes were detected in five isolates obtained from two calves, two meat samples and one patient with diarrhea. Except for the human isolate, all other isolates showed phenotypic resistance profiles predicted by the corresponding genotypic profile. Among the antibiotics tested, all four isolates were resistant to ampicillin, amoxicillin/clavulanate, and tetracycline and also showed intermediate resistance to cephalotin. The two meat isolates (isolates 26 and 27) were also resistant to streptomycin, nalidixic acid, and trimethropim-sulfamethoxazole. As we commented below, these two isolates also shared the same virulence and MLVA profiles. Considering the epidemiological link, as they were found in meat samples from the same meat processing plant, our results suggest that both isolates correspond to the same circulating strain.

Previous reports showed that integrons can be frequently detected in STEC strains and that most of the integrons can contain the *aad*A1 gene alone, or in association with the *drf* A1 gene (Morabito et al., 2002; Cergole-Novella et al., 2011). In our study, the *int*I1 gene was detected in isolate 21 which was also positive for *aadA*1 and *drf* A1 genes. Strikingly, this isolate carrying an integron and showing multiple resistances to antimicrobials was obtained from a newborn calf. Our results highlight the presence of multi-antimicrobial resistant STEC in cattle and meat in agreement with previous studies reporting the emergence and dissemination of antimicrobial resistance among STEC strains (Zhao et al., 2001; Li et al., 2011; Sasaki et al., 2012). Although antibiotic therapy is discouraged for treatment of STEC infections, the presence of antimicrobial resistant STEC strains in animals represent a risk for animal and human health. The genes coding for antimicrobial resistance could be transferred to other pathogens. Moreover, antimicrobial resistant STEC strains may have a selective advantage over other bacteria in intestines of animals under antibiotic treatments (Zhao et al., 2001). Taking into account that the same classes of antimicrobial agents are used both in humans and animals, joint efforts should be made to reduce the inappropriate use of antimicrobial agents in animals (Aidara-Kane, 2014).

In conclusion, we identified three different populations of native O26:H11 strains whose main differences were associated with genes present in mobile genetic elements. Although O26 strains harboring only *stx*_2a_ subtype have been rarely isolated from cattle and food in Europe and the United States (Pearce et al., 2006; Geue et al., 2009; Chase-Topping et al., 2012; Ison et al., 2015), *stx*_2__a_-positive strains have been an important proportion of O26:H11 strains circulating in farms in Argentina and showed to carry genes associated with high virulence, representing a potential risk for public health.

### Conflict of interest statement

The authors declare that the research was conducted in the absence of any commercial or financial relationships that could be construed as a potential conflict of interest.
